# Species-specific escape of *Plasmodium* sporozoites from oocysts of avian, rodent, and human malarial parasites

**DOI:** 10.1186/s12936-016-1451-y

**Published:** 2016-08-02

**Authors:** Alessandra S. Orfano, Rafael Nacif-Pimenta, Ana P. M. Duarte, Luis M. Villegas, Nilton B. Rodrigues, Luciana C. Pinto, Keillen M. M. Campos, Yudi T. Pinilla, Bárbara Chaves, Maria G. V. Barbosa Guerra, Wuelton M. Monteiro, Ryan C. Smith, Alvaro Molina-Cruz, Marcus V. G. Lacerda, Nágila F. C. Secundino, Marcelo Jacobs-Lorena, Carolina Barillas-Mury, Paulo F. P. Pimenta

**Affiliations:** 1 Centro de Pesquisas René Rachou-Fiocruz, Belo Horizonte, MG Brazil; 2 Fundação de Medicina Tropical Dr Heitor Vieira Dourado, Manaus, AM Brazil; 3 Instituto Leônidas e Maria Deane-Fiocruz, Manaus, AM Brazil; 4 Department of Entomology, Iowa State University, Ames, IA USA; 5 Department of Molecular Microbiology and Immunology, Johns Hopkins Bloomberg School of Public Health, Baltimore, MD USA; 6 Laboratory of Malaria and Vector Research, National Institutes of Health, Rockville, MD USA

**Keywords:** Sporozoite escape, Oocyst, Mosquito vector, *Plasmodium*, Human, Murine, Avian

## Abstract

**Background:**

Malaria is transmitted when an infected mosquito delivers *Plasmodium* sporozoites into a vertebrate host. There are many species of *Plasmodium* and, in general, the infection is host-specific. For example*, Plasmodium gallinaceum* is an avian parasite, while *Plasmodium berghei* infects mice. These two parasites have been extensively used as experimental models of malaria transmission*. Plasmodium falciparum* and *Plasmodium vivax* are the most important agents of human malaria, a life-threatening disease of global importance. To complete their life cycle, *Plasmodium* parasites must traverse the mosquito midgut and form an oocyst that will divide continuously. Mature oocysts release thousands of sporozoites into the mosquito haemolymph that must reach the salivary gland to infect a new vertebrate host. The current understanding of the biology of oocyst formation and sporozoite release is mostly based on experimental infections with *P.berghei*, and the conclusions are generalized to other *Plasmodium* species that infect humans without further morphological analyses.

**Results:**

Here, it is described the microanatomy of sporozoite escape from oocysts of four *Plasmodium* species: the two laboratory models, *P. gallinaceum* and *P. berghei*, and the two main species that cause malaria in humans, *P.viv*ax and *P. falciparum*. It was found that sporozoites have species-specific mechanisms of escape from the oocyst. The two model species of *Plasmodium* had a common mechanism, in which the oocyst wall breaks down before sporozoites emerge. In contrast, *P. vivax* and *P. falciparum* sporozoites show a dynamic escape mechanism from the oocyst via polarized propulsion.

**Conclusions:**

This study demonstrated that *Plasmodium* species do not share a common mechanism of sporozoite escape, as previously thought, but show complex and species-specific mechanisms. In addition, the knowledge of this phenomenon in human *Plasmodium* can facilitate transmission-blocking studies and not those ones only based on the murine and avian models.

## Background

Malaria remains a life-threatening disease that threatens approximately 3.4 billion people in 104 tropical countries, mainly in Africa, Asia, and South America, with an estimated 207 million cases and half a million deaths reported per year [1]. This vector-borne disease is caused by protozoa of the genus *Plasmodium,* of which *Plasmodium falciparum,* endemic to Africa, is the most prevalent species, followed by *Plasmodium vivax* in Asia and the Americas [1]. Other *Plasmodium* species infect other animal species, such as *Plasmodium gallinaceum* and *Plasmodium berghei*, responsible for avian and murine malaria, respectively [2, 3]. Many experimental studies have used *P. berghei* and *P. gallinaceum* as laboratory models to investigate the interactions between the parasites and their vectors. These two *Plasmodium* species are easily maintained in experimental animals, facilitating investigative research in laboratories [4–8].

The *Plasmodium* life cycle begins in a permissive vector when a female mosquito takes a blood meal from an infected vertebrate host that contains gametocytes, the stage of the parasite that can infect the invertebrate vector. Only a few minutes after the infective blood meal enters the midgut lumen of the susceptible mosquito, these gametocytes undergo activation to generate micro- and macro-gametes that fertilize to produce a diploid zygote. After DNA replication and the production of a 4N parasite, the zygote will differentiate into an ookinete over the next 18-24 h depending on the respective parasite species. Ookinetes are a motile form of the parasite that invade and pass through the midgut epithelium until they reach the midgut basal lamina towards the haemocoel of the mosquito. At this location, between the epithelial cells of the midgut and the basal lamina, the ookinete differentiates into a protruding rounded oocyst facing the mosquito haemocoel [8–12]. The presence of well-developed protruding oocysts in the midgut wall is indicative of infection by *Plasmodium* [13–15], and is a reliable measurement to determine the infection rate and the susceptibility of a mosquito species to a particular *Plasmodium* species. In the midgut wall, the oocysts progress to the asexual phase of multiplication known as sporogony, which is completed in approximately 1–2 weeks, the longest phase of the *Plasmodium* life cycle in the mosquito vector. Ultimately, this biological process produces thousands of sporozoites, the final form of *Plasmodium* in the vector. The sporozoites are motile sickle forms that escape from the oocysts into the mosquito hemocoel and invade the salivary gland. Once inside the salivary gland, the sporozoites are ready to be injected into a new vertebrate host via a mosquito bite, completing the *Plasmodium* life cycle in the invertebrate vector [16–18].

Completion of the *Plasmodium* life cycle in the vector requires passage through several barriers inside and outside the midgut. One important and poorly studied barrier is the exit of sporozoites from the oocyst, a critical step that allows sporozoite release into the haemolymph and subsequent invasion of the mosquito salivary gland. Knowledge of the escape mechanism of various *Plasmodium* species is largely unknown for the human malaria parasites, and only a few reports using the laboratory models have previously been published. Studies of the development of *P. berghei* oocysts using a scanning electron microscope (SEM) showed a single small hole in the oocyst wall, inside which sporozoites could be seen [19]. Sinden and Strong reported a torn oocyst from which several *P. falciparum* sporozoites had been released [20]. Meis and collaborators studying the sporogony of *P. falciparum* and *P. berghei,* reported some details of sporozoite escape and concluded that the two species showed similar mechanisms of escape, i.e., the oocysts burst and sporozoites were released into the hemocoel of the mosquito vector [21]. Although published studies have provided some details, knowledge of sporozoite escape from the oocysts of distinct *Plasmodium* species remains incomplete and is primarily based on *P. berghei*, a classical murine malarial parasite used as an experimental model in several laboratories. Moreover, most of the studies on the molecular mechanism of oocyst formation and sporozoite escape have been done using murine *P. berghei* mutant parasites, resulting in conclusions that have been generalized to human *Plasmodium* species without further morphological study.

Understanding the mechanisms of sporozoite escape in various *Plasmodium* species as well as correlations with molecular findings, may contribute to our knowledge of the parasite life cycle in the mosquito vector. Scanning electron microscopy analysis of the external side of the dissected midguts of infected mosquitos is a valuable tool for studying sporozoite escape from oocysts and has not been well explored. Here, this study provides comprehensive insight into the microanatomy of the mechanism of sporozoite escape from oocysts in four species of *Plasmodium*: the two laboratory models, avian *P. gallinaceum* and rodent *P. berghei*, and the two primary causative agents of human malaria, *P. vivax* and *P. falciparum*. It was showed that sporozoite escape is not a common biological process, as previously thought, but the mechanism is complex and species-specific.

## Methods

### Mosquito rearing

Mosquitoes of *Anopheles gambiae, Anopheles aquasalis* and *Aedes aegypti* were reared at 27 °C with 80 % humidity on 12 h light/dark cycle under insectary conditions. They were provided with 10 % sucrose solution ad libitum until 1 day before the infective blood meal, as described previously [8, 15].

### Infection of mosquitoes with *Plasmodium*

Susceptible female mosquitoes (4–5 days old) were chosen to be experimentally infected with one of the four *Plasmodium* species through a membrane feeder device at 37 °C for 30 min, as described previously [8]. *Anopheles gambiae* were infected with stage IV and V gametocytes of the cultured *P. falciparum* NF54 strain. The mature gametocytes were mixed with type O^+^ blood and offered to the mosquitoes [22–24]. *Anopheles gambiae* were also infected with *P. berghei* by direct skin feeding on infected Swiss Webster female mice with a parasitaemia level of 4–8 % and containing 2–3 gametocyte exflagellations per field when observed at 400× under a light microscope. *Aedes aegypti* were infected with *P. gallinaceum* by direct skin feeding on an infected chicken (*Gallus domesticus*) with a 10 % parasitaemia level and at least 2 % circulating gametocytes [25]. *Anopheles aquasalis* were fed on *P. vivax*-infected blood collected from patients diagnosed with malaria, as described in the Ethics statement.

### Ethics statement

For the acquisition of *P. vivax* infected human blood, patients were selected among the people visiting the Hospital at the Foundation of Tropical Medicine located in Manaus, Brazil looking for malaria diagnosis and treatment during outbreaks. Diagnosis was performed by Giemsa stained blood smear. After positive diagnosis and visualization of gametocytes, patients were interviewed and inquired about the possibility of volunteer donation of a small amount of blood for research purposes. After verbal agreement, a term of consent was first read to the potential volunteers, with detailed verbal explanation, and, after final consent, signed by the patient. After this, one 200 ml sample of venous blood was drawn from each patient and placed in heparinized tubes. Blood samples were kept under refrigeration in an icebox (at approximately 15 °C) for about 15 min, taken to the laboratory. The infected *P. vivax* blood samples were offered to mosquitoes through membrane feeder devices. Patient selection criteria were: to be *P. vivax* positive, to have about 4–8 % of circulating gametocytes as determined by the National Institutes of Health international protocols, and to consent to be part of the research consent form that was approved by the Brazilian Ministry of Health, National Council of Health, National Committee of Ethics in Research (CONEP—Approval Number 3726). All patients were treated in accordance with the Brazilian Malaria National Control Programme guidelines.

Also, mice and chickens were maintained at the Animal Care Facility of the FIOCRUZ-MG under specific pathogen-free conditions and were used in accordance to a study protocol approved by the FIOCRUZ Ethical Committee for Animal Use (CEUA; license number LW30/10). It was followed the Public Health Service Animal Welfare Assurance #A4149-01 guidelines according to the National Institutes of Health (NIH) Office of Animal Care and Use (OACU) since these studies were done according to the NIH animal study protocol (ASP) approved by the NIH Animal Care and User Committee (ACUC), with approval ID ASP-LMVR5.

### Scanning electron microscopy of infected mosquito midgut

The mosquito midguts were dissected daily, from day 8 to day 16 after the infective blood meal. The dissected midguts were fixed for 2 h in 4 % glutaraldehyde solution in 0.1 M cacodylate buffer, pH 7.2 and then post-fixed with 1 % osmium tetroxide for 2 h. The fixed samples were dehydrated using a graded acetone series, CO_2_-dried in a critical-point drying device (Emitech K850, USA) and gold-coated in a sputter coater (Emitech K550, USA) as detailed previously [26]. The samples were analyzed and imaged using a JSM-5600 scanning electron microscope (Jeol USA, Inc).

## Results

Careful comparative SEM analyses of infected midguts dissected from susceptible mosquito vectors, containing distinct *Plasmodium* species, revealed several new details of the oocyst surface and the sporozoite escape process that are unique to each *Plasmodium* species.

### Escape of *Plasmodium gallinaceum* sporozoites from oocysts

**Fig. 1:**
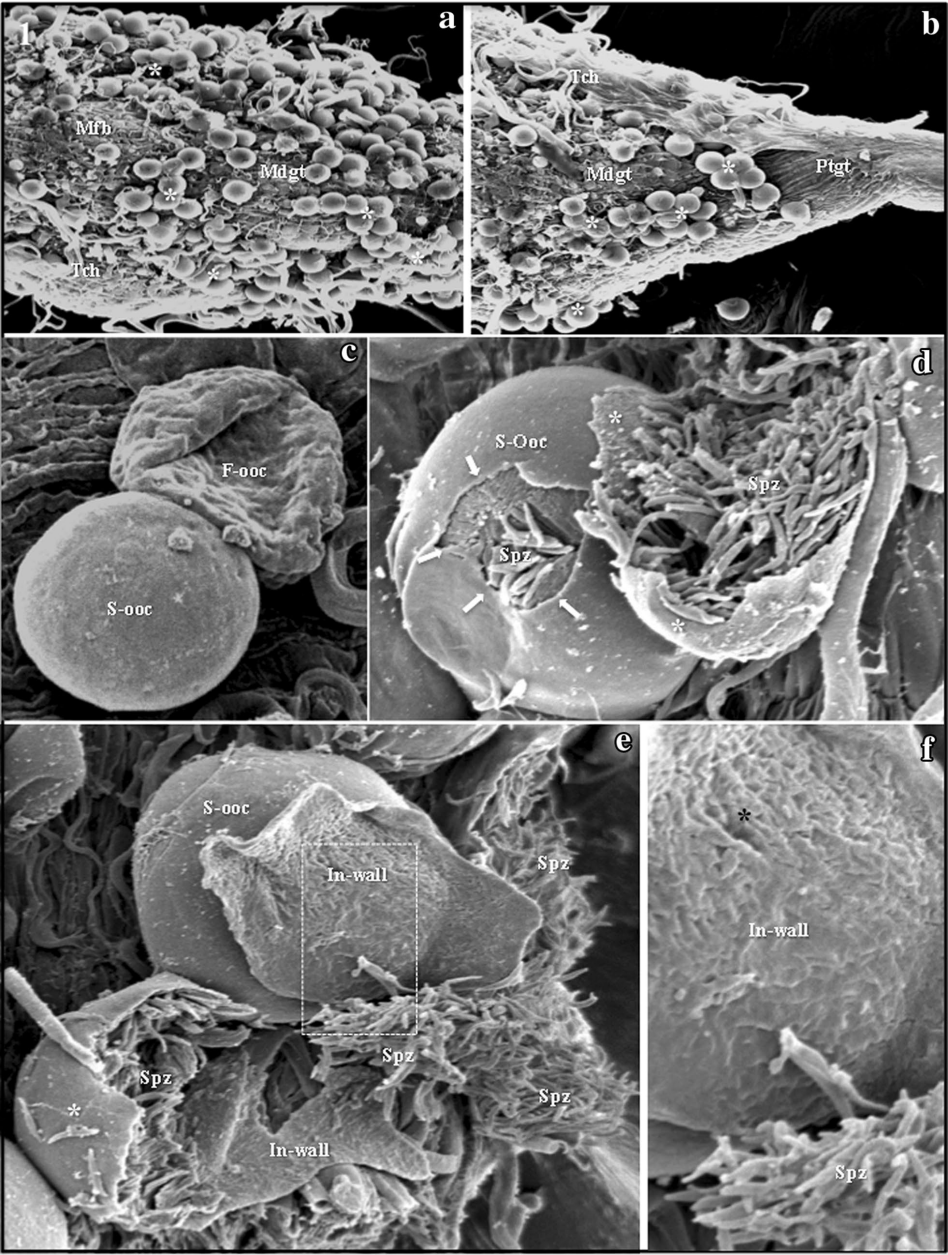
*Plasmodium gallinaceum* sporozoites escaping from oocysts. **a** and **b** Hundreds of rounded *P. gallinaceum* oocysts of similar size protruding from the external surface of the midgut (Mdgt) among the muscle fibers (Mfb) and tracheoles (Tch). Most oocysts form clusters of a few individuals (*asterisks*). All oocysts have a completely smooth surface. Magnification = ×100. **c** Two oocysts attached side by side to the midgut surface; one is completely smooth (S-ooc) and the other is flattened (F-ooc). Note the single hemocyte attached over the completely smooth oocyst wall (*arrow*). Magnification = ×1400. **d** One completely smooth oocyst (S-ooc) with a cracked wall (*arrows*) and a partly cracked oocyst (*asterisks*) showing hundreds of escaping sporozoites (Spz). Magnification = ×1700. **e** Thousands of clustered sporozoites (Spz) can be seen inside and escaping from a partly cracked oocyst (*white asterisk*). In the upper portion of the image, an empty half-shell of a broken oocyst can be seen, in which it is possible to observe details of the internal wall (In-wall). S-ooc = completely smooth oocyst. Magnification = ×1500. **f** Magnified view of the dashed area of Fig. 1d, showing the porous surface of the internal wall (In-wall) of the oocyst. Spz = clusters of escaping sporozoites. Magnification = ×4500

Magnification of dissected midguts showed hundreds of rounded avian *P. gallinaceum* oocysts on the midgut surface of the infected *Ae. aegypti*. Most of the oocysts formed small groups on the midgut surface (Fig. 1a, b). Flattened oocysts and completely smooth oocysts were observed side by side, some with haemocytes attached to the surface (Fig. 1c). On the 14th day after infection, it was possible to observe sporozoites escaping from oocysts in the dissected midguts. These dissected midguts were carefully scrutinized for the presence of oocysts, in order to observe the details of sporozoite escape. Several cracked oocysts of *P. gallinaceum* were observed at distinct stages, from some with small cracks in the surface, to some that were completely broken, exposing hundreds of escaping sporozoites (Fig. 1d–f). The completely cracked oocysts liberated thousands of sporozoites into the mosquito haemocoel (Fig. 1d, e). In empty oocyst shells, it was possible to observe the porous surface of the internal side of the oocyst wall (Fig. 1e, f).

### Escape of *Plasmodium berghei* sporozoites from oocysts

**Fig. 2:**
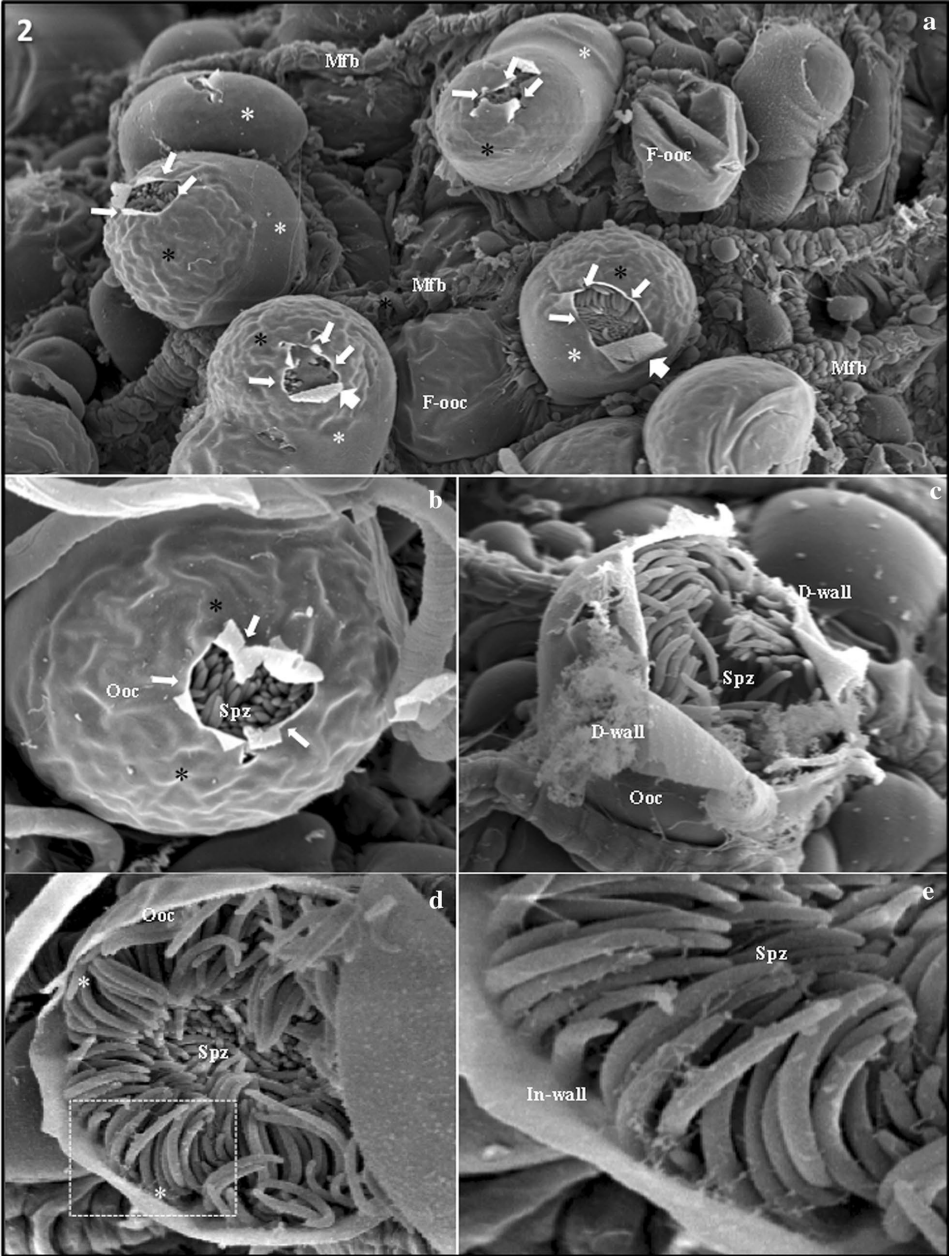
*Plasmodium berghei* sporozoites escaping from oocysts. **a** *P. berghei* oocysts protruding among muscle fibers (Mfb) on the external surface of the midgut. The surfaces of the oocysts are partly smooth (*white asterisks*) and partly wrinkled (*black asterisks*), with the exception of a few flattened oocysts (F-ooc). Several oocysts have openings on their wrinkled surfaces, which appears as if the wall has peeled away, and sporozoites can be seen inside the hole (*arrows*). Muscle fibers = Mfb. Magnification = ×1000. **b** Small opening (*arrowhead*) in the oocyst (ooc) wall showing the orderly arrangement of several sporozoites (Spz) inside the oocyst. Note the wrinkled surface of the oocyst. Magnification = ×1900. **c** Oocyst (ooc) with a large opening, approximately half-size, showing a sponge-like part of the dissolving oocyst wall (D-wall). Note cluster of sporozoites (Spz) ready to escape from the oocyst. Magnification = ×1900. **d** A single oocyst (ooc) showing a large opening with a “cap” (*asterisk*) that appears to allow sporozoite escape. Note cluster of sporozoites (spz) attached to the internal side of the oocyst wall (*arrows*). Magnification = ×1700. **e** Magnified view of the dashed area from figure **d**, showing a large opening with several orderly arranged sporozoites (Spz) attached to the internal side of the oocyst wall (In-wall). Magnification = ×4300

At 13 and 14 days after infection of *An. gambiae* with *P. berghei,* several oocysts were observed to be protruding between the muscle fibers covering the midgut surface, at different stages of rupture (Fig. 2a–d). The upper surface of these oocysts was wrinkled, and the basal surface, inserted in the midgut tissue, was smooth; it was also possible to observe some flattened oocysts (Fig. 2a). In most of the oocysts, the wall showed distinct stages of “decortication” until the sporozoites were liberated. This decortication was always present in the upper surface and in the wrinkled areas of the oocyst wall (Fig. 2a–c). In some images, it is possible to observe the advanced stages of sporozoite escape, indicated by dissolution of the oocyst wall (Fig. 2c). However, even after the oocyst had opened completely, the sporozoites remained attached to the internal side of the wall, probably until the wall was completely destroyed (Fig. 2d, e).

### Escape of *P. vivax* sporozoites from oocysts

**Fig. 3:**
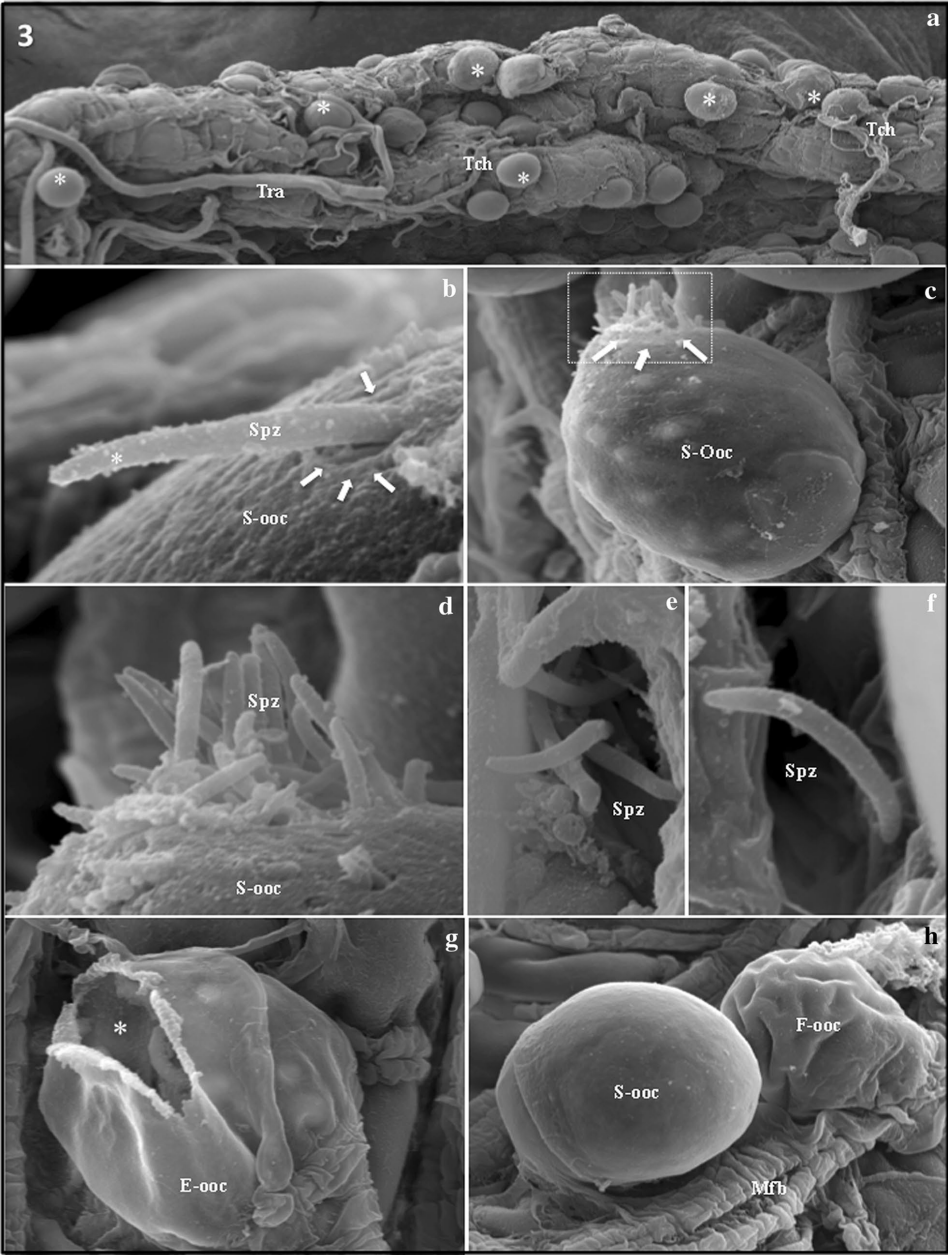
*Plasmodium vivax* sporozoites escaping from oocysts. **a** Low-power magnification of the external surface of the midgut showing protruding *P. vivax* oocysts (*asterisks*) of similar size. The rounded oocysts are arranged individually or in pairs protruding among muscle fibers (Mfb), trachea (Tra), and tracheoles (Tch) that rest on the external surface of the midgut. Magnification = ×200. **b** High-power magnification of a single sporozoite actively escaping by creating a hole (*arrows*) in the smooth oocyst (S-ooc) wall with its anterior end (*asterisk*). Note the rigid perpendicular shape of the escaping sporozoite (Spz). Magnification = ×8000. **c** and **d** Tens of grouped sporozoites (arrowheads) escaping from the lateral wall of an oocyst (S-ooc). Figure **d** is an enlarged image of the dashed area from figure **c**. Note the “rigid perpendicular shape” of the escaping sporozoites (Spz). Magnifications D = ×1400 and E = ×4000. **e** and **f** Free sporozoites (Spz) with the characteristic “comma-shape,” as seen in the mosquito hemocoel. Magnifications E = ×4100 and F = ×4300. **g** Empty oocyst (E-ooc) showing a hole through which the sporozoites escaped from the oocyst (*asterisk*). Magnification = ×1800. **h** Two side-by-side oocysts attached to the muscle fibers (Mfb) of the midgut. One smooth oocyst shows a completely stretched wall (S-ooc) and the other shows a flattened wall (F-ooc). Magnification = ×1300

The dissected midgut of the infected *An. aquasalis* revealed approximately ten to a few hundred *P. vivax* oocysts of similar size on the midgut surface. Most oocysts were isolated or in pairs and they were protruding from the basal midgut surface (Fig. 3a). Detailed analysis of infected midgut using high-magnification images allowed the observation of active escape of a single sporozoite in a rigid perpendicular position (resembling a pointing finger) at 14 days after infection. This sporozoite was forcing its way out of the oocyst by making a hole in the oocyst wall with its anterior tip (Fig. 3b), arguing that sporozoite release is directly initiated by individual or small groups of sporozoites. Additional images of 15 and 16 days after infection show the escape of a group of few sporozoites from a small hole, all with a “pointing finger” shape, indicating that they were actively forcing themselves through the wall in a striking first step to sporozoite release (Fig. 3c, d). Finally, it was observed free sporozoites with the characteristic “comma-shape” in distinct regions of the mosquito hemocoel (Fig. 3e, f). Empty oocysts, with discernible holes where sporozoites had escaped, were occasionally observed (Fig. 3g), along with some undeveloped flattened oocysts immediately adjacent to completely smooth oocysts (Fig. 3g).

### Escape of *Plasmodium falciparum* sporozoites from oocysts

**Fig. 4:**
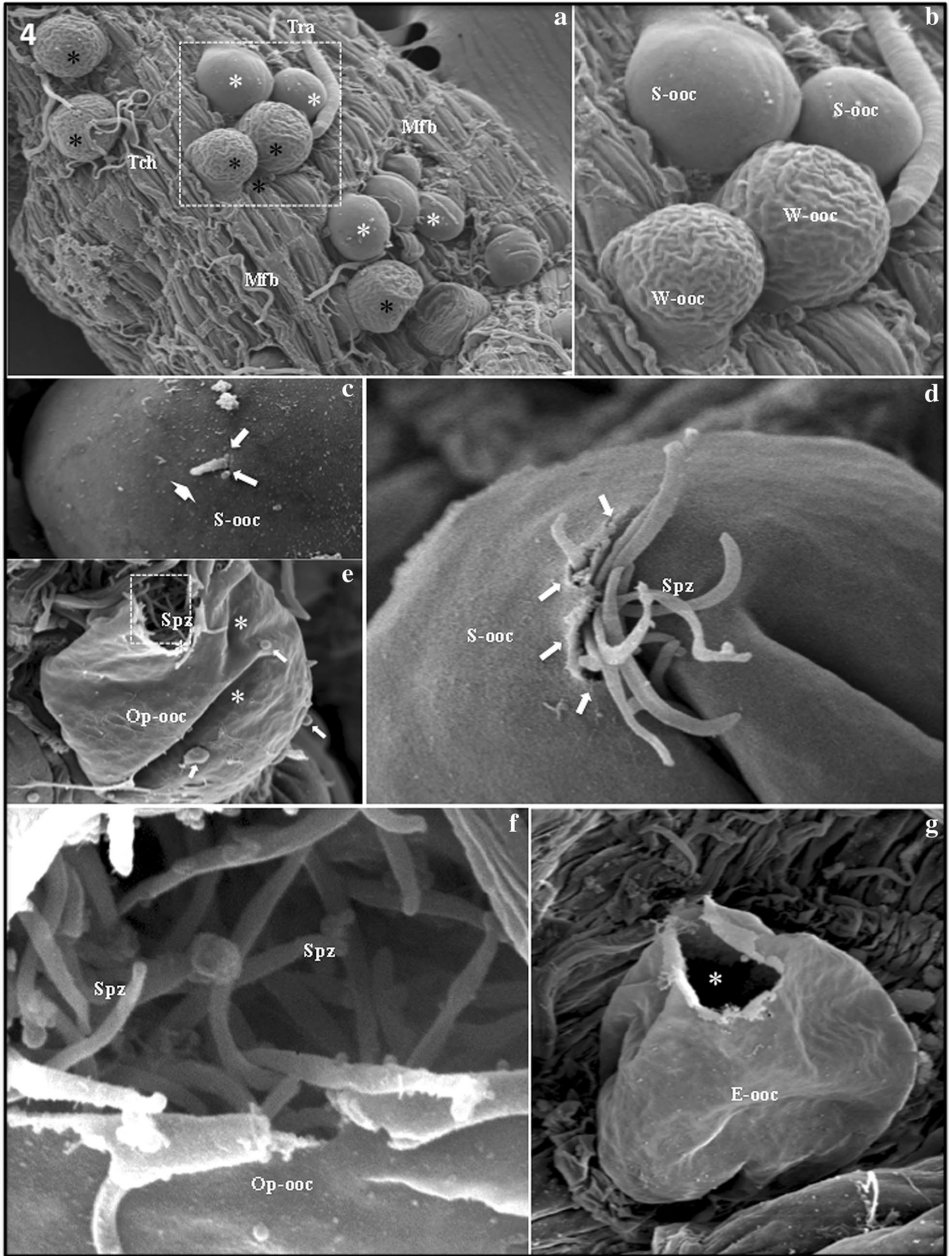
*Plasmodium falciparum* sporozoites escaping from oocysts. **a** *P. falciparum* oocysts, with numerous completely smooth oocysts (*white asterisks*) and wrinkled oocysts (*black asterisks*) protruding among muscle fibers (Mfb), trachea (Tra), and tracheoles (Tch) that rests on the external surface of the midgut. Magnification = ×350. **b** High-power magnification of the dashed area from figure **a** showing a group of four oocysts attached to the midgut. It is possible to observe surface details of two completely smooth oocysts (S-ooc) and two wrinkled oocysts (W-ooc). Magnification = ×800. **c** Enlarged image of a small portion of the initial process of a single sporozoite actively escaping from a completely smooth oocyst (S-ooc) by creating a hole (*arrows*). Note the rigid perpendicular shape of the escaping sporozoite and the anterior tip (*large arrow*) of the parasite (*large arrow*). Magnification = ×3000. **d** One completely smooth oocyst (S-ooc) with small broken areas (*arrows*) showing a group of twelve escaping sporozoites (Spz). Magnification = ×3500. **e** Flattened opened oocyst (Op-ooc) showing a lateral opening (*asterisk*) with a cluster of escaping sporozoites (Spz) that remain inside. Note hemocytes attached to the oocyst wall (*arrows*) and folded areas (*asterisks*) of the oocyst surface. Magnification = ×1300. **f** Enlarged image of the dashed area from figure **e** showing the oocyst opening and several escaping sporozoites (Spz). Note the “comma-shaped” sporozoites. Oocyst = Op-ooc. Magnification = ×6000. **g** Flattened empty oocyst (E-ooc) showing the lateral opening (*asterisk*). No sporozoites can be seen inside or around the oocyst opening. Magnification = ×1800

The dissected midgut sections of the infected *An. gambiae* revealed approximately ten to a few hundred *P. falciparum* oocysts (data not shown), most of them of similar size, located on the midgut surface. Some oocysts protruded, isolated or in groups of 4–6 individuals, on the basal midgut surface. *Plasmodium falciparum* oocysts could be classified into two distinct types according their surface: completely smooth and wrinkled surfaces (Fig. 4a, b). At 14 days after infection, detailed analysis revealed the initial process of a single sporozoite actively escaping through a unique hole, always from a completely smooth oocyst. These escaping single sporozoites also presented the “pointing finger” shape similar to those seen with *P. vivax*, leading with the anterior tip (Fig. 4c). At 13 and 14 days after infection some completely smooth oocysts showed small broken areas from which a few sporozoites were escaping. These oocysts were beginning to show folded areas on the surface (Fig. 4d). During *P. falciparum* sporozoite escape, it was possible to observe a flattened oocyst with a lateral opening, showing a cluster of escaping sporozoites inside (Fig. 4e, f). Notably, only completely smooth oocysts appeared to produce escaping *P. falciparum* sporozoites, and were never observed escaping from wrinkled oocysts.

The Table 1 shows the proportion of the distinct *Plasmodium* oocysts according to their main microanatomical aspects of their surfaces, as described in details above.

**Table 1:** Proportion of the oocysts according to their surface microanatomical details

	*P. gallinaceum* (n = 138)	*P. berghei* (n = 325)	*P. vivax* (n = 160)	*P. falciparum* (n = 162)
Flattened	7.9 %	2.1 %	11.8 %	6.8 %
Smooth	83.4 %	20 %	85 %	24.8 %
Wrinkled	–	–	–	66.4 %
Wrinkled + smooth^a^	–	58 %	–	–
Cracked	8.7 %	–	–	–
Small openings^b^	–	15.6 %	7.5 %	2.4 %
Large openings^b^	8.7 %	4 %	7.5 %	–

– Oocyst aspects not present in the *Plasmodium* species

^a^Wrinkled/smooth occysts present both characteristic aspects in their surface

^b^Small openings or large openings by our definition was related with small or large fissures considering 1/3 of the oocyst surface

## Discussion

The longest developmental stage of the *Plasmodium* life cycle in the mosquito vector is sporogony, the process of formation of thousands of sporozoites. A single parasite invades the epithelium of the midgut of a mosquito vector and remains in the gut wall for several days. This single-celled protozoan remains outside the mosquito cells, and grows into a large-lobed syncytial nucleus by mitotic division, inside a structure named the oocyst, which forms mature sporozoites. These mature sporozoites escape from the oocysts into the mosquito cavity, after which they invade the salivary gland in preparation for injection in a new vector. The duration of this stage of the *Plasmodium* life cycle varies according to the species, but usually lasts 8–14 days after the mosquito vector has ingested the infective blood meal [7–9, 13, 27].

In this study, to examine the microanatomy of sporozoite escape from oocysts of the four *Plasmodium* species, 10–20 midgut sections were dissected daily from infected mosquito vectors, 6–16 days after the infective blood meal. The midgut samples were dissected, fixed, and processed in the same laboratory, following an identical rigorous protocol to facilitate comparative analyses. The microanatomical analyses presented here clearly and accurately show the ultrastructural aspects of the oocyst surfaces and the processes of sporozoite escape. Preliminary analyses revealed that in all *Plasmodium* species, the oocysts are rounded structures that protrude individually or in small groups from the exterior of the midgut wall of the mosquito vector. However, the oocysts of the four *Plasmodium* species differ in surface features of the external wall and in the process of sporozoite escape.

In the avian parasite *P. gallinaceum,* the outer surfaces of all oocysts were completely smooth. During the process of sporozoite escape, *P. gallinaceum* oocysts were cracked, suggestive of internal forces disrupting the oocyst wall from the inside. The broken oocysts were similar to broken eggs, exposing their internal surface, with subsequent release of large groups of sporozoites into the mosquito cavity. In contrast, all murine *P. berghei* oocysts showed a hybrid surface, wrinkled on the top and smooth on the base. Compared to *P. gallinaceum, P. berghei* sporozoites appear to have a less violent mechanism of escape from the oocysts. On the upper, wrinkled surface of the oocysts, a small part of the wall begins to decorticate, creating a small opening, followed by progressive dissolution of the oocyst wall. Then, the highly structured clusters of sporozoites detach from the internal oocyst wall. In the murine and avian species of *Plasmodium,* the final steps of the sporozoite escape process, no empty oocysts were observed, distinct from the species of *Plasmodium* that infect humans. Only one comparative study of *P. gallinaceum* and *P. berghei* oocysts has been published [19]. In both *Plasmodium* species, both completely smooth oocysts and rare, wrinkled oocysts were observed, which the authors considered matured oocysts or sample preparation artifacts. Although they only showed two images, they suggested these two *Plasmodium* species have similar sporozoite escape mechanisms.

All *P. vivax* oocysts showed similar completely smooth surfaces, and in this respect, they are morphologically similar to *P. gallinaceum* oocysts. In contrast, two types of *P. falciparum* oocysts were observed: completely smooth and wrinkled oocysts. These oocysts were randomly distributed, sometimes side-by-side, in the mosquito midgut at a 50:50 ratio. Previous studies found that infected *P. falciparum* mosquitoes contained only wrinkled oocysts, but no escaping sporozoites were observed [20, 21]. The authors suggested that the wrinkled surface was characteristic of mature oocysts. However, although we also observed two types of oocysts in *P. falciparum*, sporozoites were only observed escaping from completely smooth oocysts, indicating that completely smooth oocysts contain mature sporozoites. The wrinkled oocysts may be immature oocysts or oocytes that cannot produce healthy, mature sporozoites.

The most noteworthy feature of the two human *Plasmodium* species, *P. vivax* and *P. falciparum,* is the dynamic mechanism of sporozoite escape from oocysts, distinct from that of the laboratory model *Plasmodium* species. Careful observation showed that the first signals of sporozoite escape are identical for the two human *Plasmodium* species: escape begins with a single sporozoite, in a rigid perpendicular position, forcing an exit from through the oocyst wall. The rigid perpendicular sporozoite opens a tiny hole in the oocyst wall with its anterior end. The oocyst wall is composed of two layers; the internal layer is of *Plasmodium* origin and the external thick layer that is derived from the basal lamina of the mosquito midgut [28, 29]. Moreover, in addition to allowing for growth, the capsule must have an ordered structure to allow for precursors and nutrients that support parasite growth and differentiation to enter the oocyst and metabolites to exit it [30, 31]. Subsequently, this tiny hole in the oocyst wall grows larger and allows other sporozoites to escape. Although this first step, with a single sporozoite making a tiny hole in the oocyst wall, is identical between the two species, the subsequent steps of sporozoite escape differ between *P. vivax* and *P. falciparum*. In *P. vivax,* a small group of sporozoites continue, in the same rigid perpendicular position as the first, to actively move forward to enlarge the hole in the oocyst wall. In *P. falciparum* oocysts, small groups of sporozoites escape, and individual sporozoites are flexible comma shapes, characteristic of random motion of the parasite [32–34]. A geometrical model of malaria parasite migration demonstrated that sporozoites could be modeled as self-propelled individuals that can have curved or rigid structures for motion in distinct environments [35]. This programmed rigidness and flexibility of the human *Plasmodium* sporozoites appears to act distinctly in the two species of *Plasmodium,* since it plays a role in opening the oocyst wall, allowing escape.

Molecular mechanisms related to oocyst formation and sporozoite escape have been demonstrated, mainly using mutants of murine *P. berghei*, which infects rodents, but not in *Plasmodium* species that infect humans. It is important to note that these analyses demonstrate that *P. berghei* sporozoites escape from oocysts by a process that harms the oocyst wall. The circumsporozoite (CS) protein, secreted by sporozoites, covers the internal layer of the oocyst wall [36, 37]. It was demonstrated in *P. berghei* that the disruption or deletion of some regions of the CS protein affects the formation and maturation of sporozoites, escape from the oocyst, and subsequent progression of the *Plasmodium* life cycle [38, 39]. Likewise, several other gene deletions have been described that affect *P. berghei* oocyst formation and consequent sporozoite escape: an oocyst-specific papain-like cysteine protease, known as the egress cysteine protease (ECP1), oocyst capsule protein (PbCAP380), fertilization gene (Pb GEX), lectin adhesive proteins (PbLAPs), protein kinases (PbCDLK), and nuclear forming-like protein (PbMISFIT) [40–48]. The results showed that *P. berghei* sporozoites are liberated from the oocyst by decortication and subsequent dissolution of the oocyst wall, which is consistent with a mechanism involving a proteolytic activity as has been proposed for *P. berghei* [42]. Thus, these findings indicate that proteins that act on the oocyst wall, rather than in the sporozoite, should be considered as target candidate molecules to stop transmission.

Analyses of the sporozoite escape processes in the *Plasmodium* species that infect humans clearly showed the action of the actively protunding sporozoites is dissimilar from that of murine and avian *Plasmodium* species. *Plasmodium* belongs to the phylum Apicomplexa, which is well defined by polarized extracellular stages, which contain specialized secretory organelles named micronemes and rhoptries in their anterior edge. Proteins secreted by these organelles play essential roles in attachment and invasion of target cells, as well as gliding motility, locomotion, and morphological changes [33, 49–52]. The main mode of active locomotion of the sporozoite is an actomyosin-dependent motility that is important for forward locomotion, and penetration and invasion of target cells [53]. In addition, during sporozoite motility, TRAP may coordinate the formation of contact sites and the dissociation of these contact sites from the substrate, including involvement of actin filaments [54, 55]. This raises the possibility that secretory proteins that are involved in the interplay of adhesion molecules and the invasion mechanism, well studied in invasion of host cells, can also play roles in the initial active stage that guides the escape of *P. vivax* and *P. falciparum* from the oocyst.

Careful comparative microanatomical analyses of midguts of mosquitos infected with four distinct *Plasmodium* species allowed us to make novel observations of sporozoite escape from oocysts. The key findings of this study are the morphological features that reveal for first time the mechanisms of sporozoite escape from oocysts of four *Plasmodium* species, including avian, murine, and human malarial parasites. Sporozoites of the four *Plasmodium* species exit oocysts using different mechanisms. The avian *P. gallinaceum* and murine *P. berghei* have been used as experimental models in several laboratories for infection of vertebrates and mosquito vectors. Mice infected with *P. berghei* have been used as laboratory models for human malaria [56–58] and to investigate interaction of the parasite with vectors of human malaria such as *An. gambiae* and *An. stephensi* [59–61]. It is important to state that the findings of the escape of *P. berghei* and *P. falciparum* sporozoites from oocysts were obtained from experimental infections of the same mosquito species, the *An. gambiae*. This fact suggests that the distinct mechanisms of the sporozoite escape is not dependent of the *Anopheles* species but is regulated by the *Plasmodium* species. Nevertheless, it is noteworthy to consider that these *Plasmodium* species differ in the oocyst microanatomical appearance and in the process of the sporozoite escape. Although the molecular mechanism that regulates sporozoite escape remains largely unknown, this study clearly indicates that *Plasmodium* species do not share a common mechanism, as previously thought.

## Conclusions

It was demonstrated that sporozoites of the human malarial parasites *P. vivax* and *P. falciparum* escape from the oocyst via a more active process than those of the avian and murine malarial parasites, *P. gallinaceum* and *P. berghei.* Detailed analysis showed that all four have distinct escape mechanisms. Sporozoites that infect humans actively create a hole in the oocyst wall, and are not dependent on the breakdown or dissolution of the oocyst wall for escape. These findings provide a strong basis for future studies of how to block sporozoite escape from oocysts in order to prevent transmission of malaria.
